# Age-related mitochondrial energy metabolism reprogramming occurs in granulosa cells during ovarian aging

**DOI:** 10.3389/fendo.2026.1726339

**Published:** 2026-02-02

**Authors:** Mengyu Shi, Zhicheng Jia, Xinxin Yang, Wenlong Qi, Xinwei Sun, Yongqian Li, Peixuan Wang, Ying Guo

**Affiliations:** 1 The First Clinical College, Shandong University of Traditional Chinese Medicine, Jinan, China; 2 Guang’anmen Hospital, China Academy of Chinese Medical Sciences, Beijing, China; 3 Reproductive and Genetic Center of Integrative Medicine, Affiliated Hospital of Shandong University of Traditional Chinese Medicine, Jinan, China

**Keywords:** female infertility, granulosa cells, KGN cells, mitochondrial energy metabolism reprogramming, ovarian aging

## Abstract

**Objective:**

Ovarian aging is an inevitable age-associated biological phenomenon.Enhancing clinical pregnancy outcomes in women with advanced maternal age (AMA) has emerged as a critical research priority in reproductive medicine. The current study seeks to unravel the mechanism governing mitochondrial energy metabolism reprogramming in granulosa cells (GCs) during age-associated ovarian aging.

**Methods:**

We conducted an age-stratified prospective observational study involving GC samples from 10 young infertile women (young group: 21–34 years) and 10 infertile women with AMA (AMA group: 35–42 years), all undergoing *in vitro* fertilization-embryo transfer (IVF-ET). Participants were recruited from November 2023 to November 2024. Additionally, an *in vitro* oxidative stress-induced senescence model was established using hydrogen peroxide (H_2_O_2_)-treated human ovarian granulosa-like tumor cell line (KGN cells) to further investigate metabolic disturbances and mitochondrial reactive oxygen species (mtROS) levels in senescent GCs.

**Results:**

High-resolution targeted metabolomics revealed 25 statistically significant metabolite alterations in ovarian GCs, indicating profound dysregulation of core energy metabolism pathways—particularly oxidative phosphorylation (OXPHOS), glycolysis, and the tricarboxylic acid (TCA) cycle. Compared to the young group, the AMA group exhibited upregulated glycolytic metabolites alongside downregulated OXPHOS and TCA cycle intermediates. These findings were further validated in an H_2_O_2_-induced KGN cells senescence model, where treated cells demonstrated: (1) increased senescence-associated β-galactosidase (SA-β-gal) activity, (2) elevated extracellular acidification rate (ECAR) and lactate (Lac) production, (3) reduced oxygen consumption rate (OCR), (4) depleted glucose and pyruvate(Pyr) pools, and (5) heightened mtROS generation relative to control group.

**Conclusions:**

Collectively, our research demonstrates that GCs undergo mitochondrial energy metabolism reprogramming, characterized by a metabolic shift from OXPHOS to glycolysis, during ovarian aging. These observations suggest that age-associated glycometabolic perturbations may represent a novel therapeutic target for infertility in women with AMA.

## 1 Introduction

Against the backdrop of evolving societal trends and shifting reproductive preferences, optimizing assisted reproductive technology for women with AMA presents a significant challenge in reproductive medicine. Maternal age is a critical determinant of female fertility, with 35 years established as the threshold for clinically relevant decline in reproductive potential. The young group included infertile women aged 21–34years, while the AMA group consisted of those aged ≥35 years (1). AMA women exhibit marked deterioration in ovarian reserve parameters, characterized by diminished oocyte quantity and quality, resulting in reduced natural conception rates and elevated miscarriage risks. This age-related ovarian aging—recognized as a primary driver of systemic female aging and a key contributor to age-associated infertility—is mediated through multifactorial mechanisms including mitochondrial dysfunction, chromosomal instability, environmental exposures, and comorbidities that collectively deplete and compromise the ovarian follicular pool (2–4). Crucially, mitochondrial dysfunction has been extensively implicated in cellular senescence (5), with age-dependent bioenergetic decline demonstrated as a hallmark of ovarian aging (6).

Mitochondria serve as the central metabolic hub of eukaryotic cells, orchestrating core bioenergetic processes including glucose, lipid, and amino acid metabolism (7). Their functional integrity and homeostatic regulation are essential for cellular viability (8). Mitochondrial metabolic reprogramming denotes the dynamic adjustment of metabolic fluxes through key pathways, enabling cells to adapt bioenergetic output to environmental demands by modulating intracellular metabolites (9). This reprogramming is intrinsically linked to biological processes such as cellular senescence, with glucose and Lac metabolism demonstrating well-established crosstalk with aging, oncogenesis, inflammation, and immunity (10). In senescent cells, mitochondrial reprogramming alters carbohydrate, lipid, and amino acid utilization—notably characterized by glycolytic upregulation, mitochondrial remodeling, and TCA cycle rewiring (11). The trajectory of glucose metabolic shifts depends critically on cell type, tissue origin, *in vivo* versus *in vitro* contexts, and specific senescence triggers. While mitochondrial metabolic reprogramming represents a promising frontier in reproductive research, its role in ovarian aging remains insufficiently explored in both mechanistic depth and pathophysiological breadth.

As a pivotal component supporting oocyte development, the functional status of GCs directly influences oocyte quality and quantity (12). Previous studies have demonstrated that oocytes cannot directly utilize glycolysis for energy supply; instead, they primarily depend on Pyr and Lac generated via glycolysis in ovarian GCs, which serve as energy substrates to fuel follicle growth, development, and maturation through mitochondrial OXPHOS (13). A correlation has been established between ovarian GCs and clinical outcomes in elderly infertile patients undergoing IVF-ET. Metabolomics aims to quantify a broad range of small molecules under physiological stimuli or disease states, while targeted metabolomics, leveraging knowledge accumulated from years of biochemical research, offers high sensitivity and reproducibility, enabling the measurement of defined chemical entities and biochemically annotated metabolite groups (14). In the present study, we sought to characterize the targeted metabolomic profiles of ovarian GCs between infertile women with AMA and their younger counterparts, and to investigate changes in relevant indicators using an *in vitro* model of senescent KGN cells.

## 2 Materials and methods

### 2.1 Study population

Patients were recruited from the Comprehensive Research Center for Reproduction and Genetics, Affiliated Hospital of Shandong University of Traditional Chinese Medicine, between November 2023 and November 2024. This prospective study enrolled 60 infertile patients undergoing IVF-ET treatment at our center, who were stratified into two groups: the young group (21 ≤ age ≤ 34 years, n = 30) and the AMA group (35 ≤age ≤ 42 years, n = 30). The inclusion criteria, based on international consensus, were as follows: (1) women aged 21–42 years planning to undergo IVF-ET; (2) patients eligible for IVF-ET due to tubal factors or male-factor infertility; (3) a history of regular menstrual cycles; (4) provision of written informed consent after comprehensive counseling.

The exclusion criteria included: (1) Polycystic ovary syndrome, diagnosed according to the 2003 Rotterdam criteria and the 2018 evidence-based guideline from the European Society of Human Reproduction and Embryology and the American Society for Reproductive Medicine, requiring at least two of the following features: oligo-/anovulation, clinical or biochemical hyperandrogenism, or polycystic ovarian morphology on ultrasound, after exclusion of other disorders with similar presentations (e.g., thyroid dysfunction, hyperprolactinemia) (15, 16); (2) Endometrial polyps, identified per the Society of Obstetricians and Gynaecologists of Canada guidelines, defined by transvaginal ultrasonography as a homogeneous hyperechoic or isoechoic intracavitary mass, sometimes with a visible stalk and possible displacement of the endometrial line, or confirmed by saline infusion sonography as a well-defined intracavitary protrusion surrounded by fluid, or hysteroscopically as a smooth-surfaced polypoid structure attached by a pedicle (17); (3) Endometritis, assessed using a combination of hysteroscopic and histopathologic criteria, with hysteroscopic features based on the 2019 International Consensus including strawberry-like spots, focal endometrial hyperemia, micropolyps, stromal edema, or punctate hemorrhages, and histologic confirmation relied on the identification of plasma cell infiltration within the endometrial stroma. This was further quantified by immunohistochemical staining for CD138, applying the College of American Pathologists guideline threshold of ≥5 CD138-positive plasma cells per 10 high-power fields as the definitive diagnostic criterion (18); (4) Uterine fibroids, diagnosed in accordance with the International Federation of Gynecology and Obstetrics classification system, with the presence of one or more fibroids confirmed by transvaginal ultrasound regardless of location, number, or size (19); (5) patients with a history of 3 or more transfer cycles (including fresh and frozen-thawed embryo transfers) with 4 or more high-quality embryos transferred without achieving pregnancy; (6) patients with a body mass index (BMI) > 28 kg/m²; (7) patients requiring pre-implantation genetic diagnosis; (8) patients with comorbidities such as severe systemic diseases, physical or psychological disorders, or thyroid/adrenal dysfunction.All participant exclusions were based on definitive diagnoses made by certified specialists under blinded conditions. Ultrasound and histopathological evaluations were independently reviewed by a certified sonographer or pathologist, respectively. Clinical diagnoses were adjudicated by two board-certified reproductive gynecologists according to established guideline criteria. In cases of discrepancy between the two initial reviewers, a third specialist in the same field was consulted for final arbitration.

### 2.2 Ethical approval

Ethics approval and consent to participate: This study follows the Helsinki Declaration (20). The study was approved by the Reproductive Ethics Committee of the Affiliated Hospital of Shandong University of Traditional Chinese Medicine (NO:SDTCM/E2867-36). All participants provided written informed consent before sample collection.

### 2.3 Study variables

At baseline, we collected the following covariates from infertile patients undergoing *in vitro* fertilization/intracytoplasmic sperm injection (IVF/ICSI) treatment: age, duration of infertility, BMI, basal follicle-stimulating hormone (FSH), basal luteinizing hormone (LH), basal estradiol (E_2_), basal progesterone (P), anti-Müllerian hormone (AMH), as well as the number of retrieved oocytes, Mature oocytes at metaphase II (MII) oocyte count: MII stage, characterized by uniform cytoplasm and extrusion of the first polar body, representing fertilization-competent oocytes in assisted reproductive technology cycles (Istanbul/ESHRE consensus). 2-pronuclei (2PN) count: normally fertilized oocytes (Istanbul/ESHRE consensus). Blastocyst count: embryos developed to blastocyst stage by day 5/6 (Gardner & Schoolcraft system). High-quality blastocysts: blastocysts graded 3–6 with inner cell mass and trophectoderm both ≥ B (Gardner & Schoolcraft criteria). MII oocyte rate: (MII oocytes/total oocytes retrieved) × 100%.2PN fertilization rate: (2PN zygotes/MII oocytes) × 100%. Blastocyst formation rate: (blastocysts/2PN zygotes) × 100%. Good-quality blastocyst rate: (good-quality blastocysts/total blastocysts) × 100%. For metabolomic analysis, 10 participants were randomly selected from each of the young and AMA groups, which initially comprised 30 patients per group.

All participants underwent IVF/ICSI treatment using a GnRH-ant protocol. Recombinant human FSH (Gonal-F, Merck Serono, Switzerland) is administered on the second or third day of the menstrual cycle at a dose of 100–300 IU per day, depending on the woman’s age, FSH, antral follicle count, and AMH. During controlled ovarian stimulation, participants were monitored for follicular recruitment and growth and endometrial thickness by serial transvaginal ultrasound and blood hormone tests, including E_2_, P and LH plasma levels. The dose of FSH may be increased or decreased according to the follicular development of the patient during controlled ovarian stimulation, within the range of 50 IU. Cetrorelix (Merck Serono, Switzerland) of 0.25 mg/day was initiated until the trigger day, when the dominant follicle diameter was ≥ 14 mm, E_2_ ≥ 400 pg/ml. Human chorionic gonadotropin (hCG, Lizhu, Zhuhai, China) or GnRH agonist (triptorelin acetate; France) combined hCG (dual trigger) was administered to trigger the maturation of oocytes when there were three follicles measuring 18 mm or more in diameter. Oocyte pick-up was performed by transvaginal ultrasound-guided needle aspiration 35–36 hours following triggering, followed by standard IVF/ICSI as previously reported. Follicular fluid (FF) was collected from individual follicles during oocyte retrieval. For each patient, FF from all aspirated follicles (including those containing mature and immature oocytes) was pooled to form a single sample representative of the individual (no inter-patient pooling), representing one independent biological replicate. GCs were isolated from the pooled FF of each patient using density gradient centrifugation (Percoll), a standard technique for isolating specific cell types. Purified GCs were stored at -80°C until further analysis.

### 2.4 Targeted metabolomic profiling of GCs

Frozen samples stored at −80°C were thawed at 4°C. Then, 200 μL of pre-cooled methanol and 10 μL of 10 mM succinic acid−D6 (internal standard) were added, and the mixture was homogenized for 2 min. Next, 400 μL of pre-cooled chloroform was added, followed by an additional 2 min of homogenization. After vortexing for 10 min, 100 μL of deionized water was added, and the mixture was shaken for another 10 min. The samples were centrifuged at 14,000×g and 10°C for 20 min. A 200 μL aliquot of the upper layer (with chloroform as the lower phase) was collected, vacuum-dried at room temperature or dried under a nitrogen stream, and stored at −80°C. For Liquid Chromatography–Mass Spectrometry analysis, the dried samples were reconstituted in 100 μL of acetonitrile–water (1:1, v/v), vortexed for 30 s, and centrifuged at 14,000×g and 10°C for 15 min. The supernatant was collected for analysis, and the remaining material was stored at −80°C.

Chromatographic separation was performed on an Agilent 1290 Infinity Liquid Chromatography ultra-high performance liquid chromatography system. The chromatographic conditions were as follows: column temperature, 35°C; flow rate, 300 μL/min; injection volume, 2 μL; mobile phase A, 50 mM ammonium acetate in water (adjusted with 1.2% ammonium hydroxide); mobile phase B, acetonitrile containing 1% acetylacetone. The gradient elution program was: 0–1 min, 70% B; 1–10 min, 60%–70% B; 10–12 min, 30%–60% B; 12.1–15 min, 30% B; 15–15.5 min, 30%–70% B; 15.1–22 min, 70% B. Quality control (QC) samples were inserted at regular intervals throughout the sample sequence to monitor system stability and reproducibility. In addition, a standard mixture of target analytes was analyzed for retention time calibration.Mass spectrometry was carried out using a 5500 QTRAP mass spectrometer operated in negative ion mode. The electrospray ionization source parameters were set as follows: source temperature, 450°C; ion source gas 1, 45 psi; ion source gas 2, 45 psi; curtain gas, 30 psi; ion spray voltage, −4500 V. Multiple reaction monitoring was employed for the detection of target ion pairs.

To ensure technical reproducibility throughout the metabolomic profiling process, the following measures were implemented: (1) QC samples were analyzed at regular intervals 377 to monitor instrument stability. The relative standard deviations 378 (RSDs) of all metabolites in the QC samples were below 10% , indicating good 380 repeatability; (2) each sample was analyzed in three technical replicates, and the mean peak area was used for subsequent statistical analysis to minimize the influence of random detection variability.

### 2.5 Culture and grouping of the KGN cells

The KGN cells, a human ovarian granulosa-like tumor cell line, were employed to mimic the aging phenotype, as oxidative stress-induced GC apoptosis is a key mechanism underlying follicular atresia. The KGN cells were commercially obtained from Shandong Unspiral Biological Company (Shandong, China) and cultured in DMEM supplemented with 10% fetal bovine serum, 100 U/mL penicillin, and 100 U/mL streptomycin at 37°C in a 5% CO_2_ atmosphere. Cell proliferation was assessed using a Cell Counting Kit-8 (CCK-8; C0039, Beyotime, China): KGN cells were seeded into 96-well plates at a density of 10,000 cells per well. The cells were subsequently exposed to serially diluted H_2_O_2_ (0, 50, 100, 200, 400, and 600 μmol/L) for the indicated time periods (0, 2, 4, 6, and 24 h). The final volume of H_2_O_2_ solution in each well was kept below one-tenth of the total suspension volume. After intervention, 10 μL of CCK-8 solution was added to each well, followed by 4 h of incubation; absorbance was measured at 450 nm using a microplate reader (Thermo Fisher, Multiskan Sky). The experimental design was configured with three biological replicates for every dose-time condition, from which a complete dataset of 90 independent data points was obtained.Based on the CCK-8 results, treatment with 50 μM H_2_O_2_ for 2 h was selected to establish the oxidative stress-induced senescence model of KGN cells. KGN cells in the logarithmic growth phase were subsequently divided into the control group and H_2_O_2_ group.

#### 2.5.1 SA-β-gal staining

Following a 2-hour stimulation period, cells were subjected to SA-β-gal staining according to the following protocol: air-dried monolayers were fixed at room temperature for 10–15 minutes using SA-β-gal fixative, followed by three 5-minute PBS washes. The samples were then incubated in freshly prepared working solution [containing 940 μL Staining Solution A (Servicebio #G1073-2), 10 μL Solution B (#G1073-3), and 50 μL X-Gal (#G1073-1) with a final X-Gal concentration of 1 mg/mL in DMF, pH 6.0] at 37°C for 16–18 hours in darkness. After staining, samples underwent two 5-minute PBS washes, two 1-minute rinses with deionized water, counterstaining with 0.1% Nuclear Fast Red for 3 minutes, dehydration through an ethanol series (70%, 95%, and 100%, 5 minutes each), clearing in xylene (two 5-minute changes), and final mounting with neutral balsam. For experimental setup, both control and H_2_O_2_-treated groups included three biological replicates (n=3), with cells seeded at a density of 2×10^5^ cells per well in 6-well plates. SA-β-gal-positive cells were identified by the presence of cytoplasmic blue chromogen deposition under bright-field microscopy, with nuclei appearing red due to counterstaining.

#### 2.5.2 Seahorse XF

Metabolic profiling was performed using the Seahorse XFe24 Analyzer (Agilent Technologies) to assess mitochondrial respiration and glycolytic function via the OCR and ECAR respectively. The Seahorse XF Cell Mito Stress Test Kit (Cat. No. 103015-100) and the XF Glycolysis Rate Assay Kit (Cat. No. 103344-100) were employed according to the manufacturer’s protocols. Target cells were seeded into Seahorse XF24 cell culture microplates (Agilent Technologies, Cat. No. 100777-004) at a density of 1×10^4^cells per well. After allowing for adhesion for 2–4 hours in a 37°C, 5% CO_2_incubator, the cells were exposed to 50 μmol/L H_2_O_2_ for 2 hours (Untreated cells served as the control group). Before the assay, the original medium was replaced: 175 μL was aspirated, cells were washed twice with 600 μL of Seahorse XF assay medium, and then 450–525 μL of fresh assay medium was added. The plate was equilibrated for 1 hour in a non-CO_2_ incubator prior to measurement. For the Mito Stress Test, oligomycin, FCCP, rotenone, and antimycin A were prepared as 2.5 mmol/L stock solutions. During the assay, each compound was sequentially injected through the designated ports of the analyzer to achieve a final working concentration of 1 μmol/L in the measurement well. For the Glycolysis Rate Assay, glucose (100 mM stock), oligomycin (100 μM stock), and 2-deoxyglucose (500 mM stock) were injected sequentially. The analyzer was calibrated before each run using a Seahorse XF Calibration Plate, and background correction was applied using designated background wells. Experiments included three biological replicates (independent cultures) per condition, each with three technical replicates (wells). Following the Seahorse assay, cells in each well were lysed for total protein quantification. Briefly, the assay medium was removed, and cells were washed once with cold PBS and lysed on ice with 100 μL of RIPA buffer containing protease inhibitors. After centrifugation at 12,000 ×g for 15 min at 4°C, the supernatant was collected. The total protein concentration was determined using a BCA protein assay kit (Pierce™) according to the manufacturer’s instructions. A standard curve was generated using bovine serum albumin. The final OCR and ECAR values were normalized to the total protein content (μg).

#### 2.5.3 Assays for Lac, Pyr, and glucose levels

Intracellular levels of Lac, Pyr, and glucose were quantified using specific commercial assay kits according to the manufacturers’ instructions (Lac: Canspec Scientific, #ADS-W-T009-48; Pyr: #ADS-W-S014; Glucose: #ADS-W-TDX002). Briefly, approximately 5×10^6^ cell pellets were resuspended in 1 mL of the corresponding extraction buffer (for Lac and Pyr) or PBS (for glucose). Cell lysis was performed via ice-bath sonication (200 W power, 3 s sonication with 10 s intervals, repeated 30 cycles). The resulting lysates were centrifuged under specified conditions (Lac: 4°C, 12,000 ×g, 10 min; Pyr: room temperature, 12,000 ×g, 10 min; glucose: 4°C, 12,000 ×g, 10 min), and the supernatants were collected for subsequent analysis. The concentrations of Lac, Pyr, and glucose were determined using a microplate reader at wavelengths of 450 nm, 340 nm, and 520 nm, respectively. The final data were normalized to the total protein concentration for analysis.

#### 2.5.4 Intramitochondrial mtROS levels

Intracellular mitochondrial ROS levels were quantified using a commercial ROS assay kit (Canspec Scientific, Cat. #ADS-W-FM016). Briefly, after trypsinization and PBS washing, cells were resuspended in serum-free medium containing 10 μM DCFH-DA probe at a density of 2×10^6^ cells/mL. The suspension was protected from light and incubated at 37°C under 5% CO_2_ for 25 minutes. Subsequently, the cells were washed twice with PBS to thoroughly remove any non-internalized probe and finally resuspended in PBS to form a single-cell suspension. Fluorescence intensity was immediately measured using a NovoCyte flow cytometer (Agilent) with excitation and emission wavelengths set at 488 nm and 530 nm, respectively. The results are expressed as mean fluorescence intensity and were normalized to the total protein concentration for statistical analysis.

### 2.6 Data analysis

Statistical analyses were performed using GraphPad Prism software. Data are presented as mean ± standard deviation (SD) or as mean with 95% confidence intervals (CI), as indicated. A p -value of < 0.05 was considered statistically significant. For targeted metabolomics, group differences in individual metabolites were analyzed using independent samples t-tests. The Benjamini-Hochberg procedure was applied to control the false discovery rate (FDR) across all detected metabolites, with statistical significance set at q < 0.05. Technical reproducibility was ensured by QC samples, with the RSD for all metabolites being < 10%. In Seahorse XF analyses, OCR and ECAR data were normalized to total cellular protein content and treated as repeated measures. Statistical analysis was performed using two-way repeated-measures analysis of variance (ANOVA) to examine the main effects of group, time, and their interaction, with estimated marginal means and their 95% CIs reported. Cell proliferation (CCK-8 assay) data across a full dose-time grid were assessed by two-way ANOVA to examine the main effects of Dose and Time and their interaction, followed by Tukey’s *post hoc* test for multiple comparisons. Group differences in SA-β-gal staining were compared using Welch’s corrected unpaired t -tests, with effect sizes reported as Cohen’s d. Measurements of lactate, glucose, pyruvate, and mitochondrial ROS were compared between groups using Welch’s t -test, and the resulting p -values were adjusted for multiple comparisons using the Benjamini-Hochberg FDR correction. As this was a prospective study and given the potential confounding between clinical parameters (e.g., age, ovarian reserve) and metabolic outcomes, all group comparisons were performed without adjustment for these covariates; this is acknowledged as a study limitation.

## 3 Results

### 3.1 Comparative baseline characteristics: a cohort of 20 IVF-ET patients stratified by age (Young vs. AMA)

The clinical characteristics of the patients are shown in **Table 1**. The baseline characteristics were reported based on the 10 patients randomly selected from each group for metabolomic analysis.The standardized mean difference (SMD) analysis revealed substantial imbalances between the Young and AMA groups across most key parameters, consistent with expected age-related declines in reproductive potential. Apart from negligible differences in BMI and basal LH, P, and E2 levels (all |SMD| < 0.5), large effect sizes (|SMD| ≥ 0.8) were observed for age, duration of infertility, ovarian reserve markers (AMH, basal FSH), oocyte acquired, and all embryological outcomes (MII, 2PN, blastocyst, and good-quality embryo numbers), as well as laboratory efficiency rates (MII, blastocyst formation, and good quality embryos rates). The fertilization rate showed a moderate difference (SMD = 0.54). These quantified imbalances provide essential context for the subsequent age-stratified metabolomic investigations.

**Table 1:** Baseline characteristics of patients.

Patient Information	Young group (N=10)	AMA group (N=10)	SMD
Age (years)	28.30 ± 2.83	40.90 ± 1.29	5.73
Duration of infertility (years)	2.10 ± 1.21	4.30 ± 2.26	-1.26
BMI (kg/m2)	22.23 ± 3.36	22.41 ± 3.10	-0.06
Basal FSH (mIU/ml)	5.53 ± 1.45	8.41 ± 2.89	-1.26
Basal LH (mIU/ml)	4.52 ± 2.43	4.32 ± 1.97	0.09
Basal E2 (pg/ml)	75.68 ± 83.58	45.55 ± 26.71	0.49
Basal P (ng/ml)	0.68 ± 0.56	0.56 ± 0.52	0.23
AMH(ng/mL)	3.80 ± 1.57	1.10 ± 0.54	2.23
Oocytes acquired	11.80 ± 4.02	3.50 ± 1.72	2.69
Number of MII	10.20 ± 4.21	2.60 ± 1.58	2.39
Number of 2PN	9.40 ± 4.17	2.40 ± 1.58	2.22
Number of blastocysts	5.40 ± 3.53	0.60 ± 1.07	1.85
Number of good quality embryos	1.80 ± 1.55	0.10 ± 0.32	1.52
IVF Laboratory outcomes (efficiency ratios, %)			
MII rate	84.60	64.30	0.94
Fertilization rate (2PN/MII)	90.19	74.17	0.54
Blastocyst formation rate	52.94	16.67	1.12
Good-quality blastocyst rate	30.56	3.33	1.3018

(Data are presented as mean ± SD).

### 3.2 Targeted metabolomics reveals mitochondrial metabolic reprogramming in aging ovarian GCs

**Figure 1:**
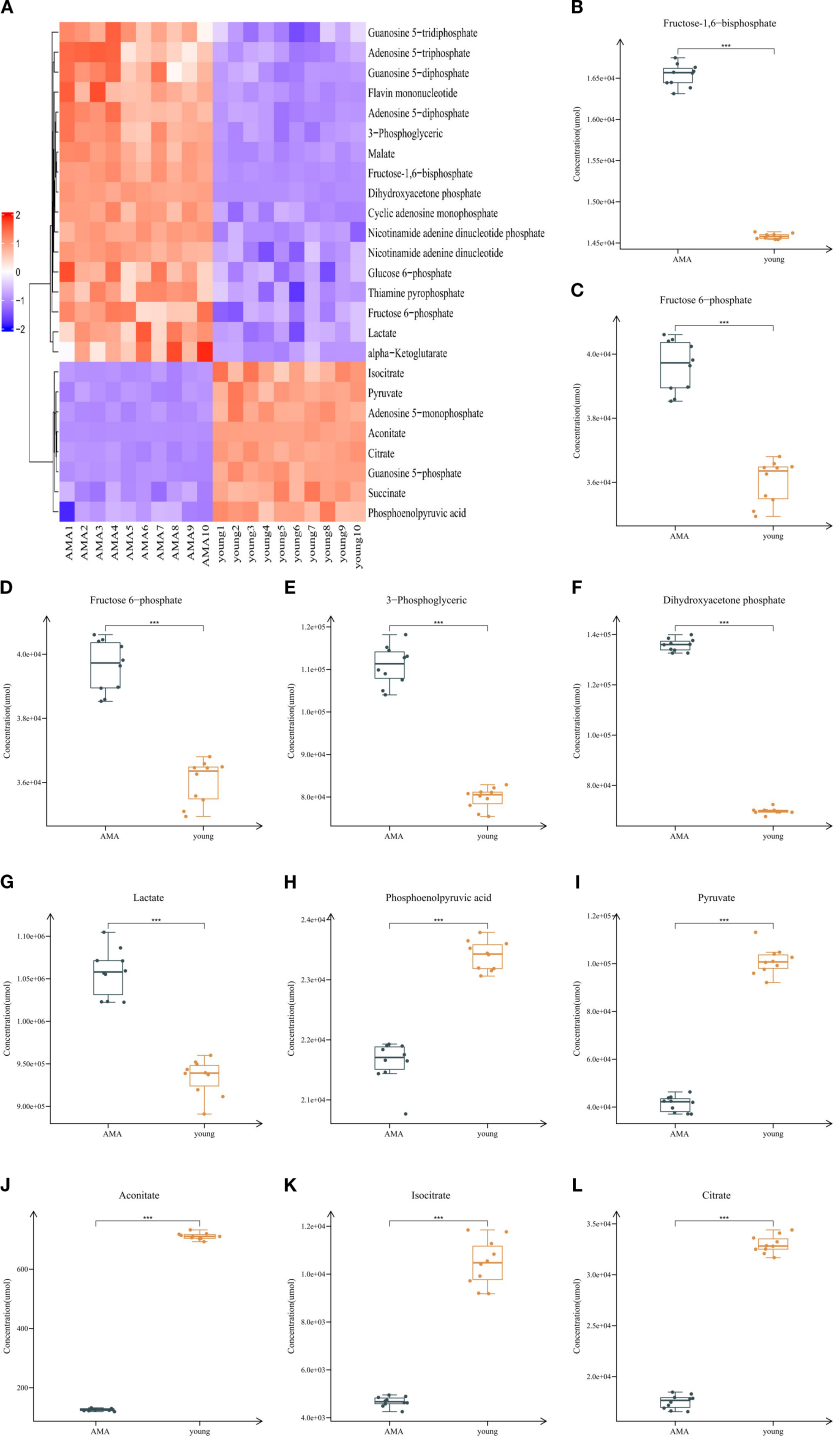
Metabolic divergence between young and AMA groups. **(A)** Heatmap displaying metabolic differences between the young group and AMA group. **(B-L)** Box plots demonstrating differential metabolites between the young group and AMA group. (Data are presented as mean ± SD, *p < 0.05, **p < 0.01, ***p < 0.001).

Targeted metabolomics enables the assessment of glucose metabolism levels in infertile women with AMA. In this study, liquid chromatography-mass spectrometry was used to investigate the targeted glucose metabolic profiles of GCs derived from infertile patients undergoing IVF/ICSI. The results revealed distinct differences in the metabolic profiles of GCs between the AMA group and the young group. A total of 32 differential metabolites were identified between the two groups, which were associated with pathways including OXPHOS, glycolysis, the TCA cycle, and gluconeogenesis; after FDR correction, 25 of these metabolites remained statistically significant (**Table 2**). Compared with the young group, 17 metabolites were up-regulated and 8 were down-regulated in the AMA group (**Figure 1A**). The up-regulated metabolites included adenosine 5’-diphosphate, ATP, cyclic adenosine monophosphate, flavin mononucleotide, fructose 1,6-diphosphate (**Figure 1B**), fructose 6-phosphate (**Figure 1C**), glucose 6-phosphate (**Figure 1D**), guanosine 5’-diphosphate, guanosine 5’-triphosphate, alpha-ketoglutarate, malate (Mal) nicotinamide adenine dinucleotide (NAD^+^), nicotinamide adenine dinucleotide phosphate, 3-phosphoglyceric acid (**Figure 1E**), dihydroxyacetone phosphate (**Figure 1F**), Lac (**Figure 1G**), and thiamine pyrophosphate; the down-regulated ones included adenosine 5’-monophosphate, guanosine 5’-monophosphate, phosphoenolpyruvic acid (**Figure 1H**), Pyr (**Figure 1I**), aconitate (Aco) (**Figure 1J**), isocitrate (Ici) (**Figure 1K**), citrate (Cit) (**Figure 1L**), and succinate. Specifically, glycolytic metabolites such as fructose-1,6-bisphosphate, fructose 6-phosphate, glucose 6-phosphate, 3-phosphoglyceric acid, dihydroxyacetone phosphate, and Lac were increased in the AMA group, while phosphoenolpyruvic acid and Pyr were decreased. Additionally, compared with the young group, the AMA group exhibited increased glucose uptake and Lac production, along with decreased Pyr production. Regarding the TCA cycle, the levels of related metabolites including Aco, Ici, and CA were reduced in the AMA group. We hypothesize that GCs from infertile women with AMA undergo a reprogramming of mitochondrial energy metabolism, shifting from OXPHOS to aerobic glycolysis. This metabolic shift leads to enhanced glycolysis accompanied by reduced OXPHOS and TCA cycle activity, which may influence epigenetic changes and thereby promote the aging process.

**Table 2:** 25 metabolic changes in the young group and AMA group.

Metabolite	Young group	AMA group	QC RSD	Fold change	p-value	Up/Down	q-value
Aco	711.00 ± 10.97	126.07 ± 4.06	0.02	0.18	9.61221E-30	↓	2.40305E-28
AMP	1447.26 ± 87.21	560.92 ± 35.05	0.03	0.39	8.90923E-17	↓	2.22731E-15
ADP	1436.95 ± 24.71	1770.48 ± 54.72	0.04	1.23	8.9158E-13	↑	2.22895E-11
ATP	2410.47 ± 5.62	246.75 ± 16.52	0.01	1.02	2.72629E-08	↑	6.81572E-07
Cit	32978.46 ± 865.10	17519.91 ± 678.75	0.03	0.53	7.39936E-20	↓	1.84984E-18
cAMP	36.25 ± 1.84	53.40 ± 1.62	0.06	1.47	1.70117E-14	↑	4.25293E-13
DHAP	69801.67 ± 1176.41	135786.75 ± 2478.11	0.07	1.95	4.92856E-24	↑	1.23214E-22
FMN	228.49 ± 0.70	243.04 ± 3.03	0.02	1.06	1.58233E-11	↑	3.95583E-10
FBP	14581.60 ± 32.56	16536.80 ± 136.01	0.01	1.13	8.25208E-20	↑	2.06302E-18
F6P	36011.38 ± 675.84	39619.41 ± 805.32	0.05	1.10	2.49989E-09	↑	6.24972E-08
G6P	80322.31 ± 1736.73	89788.53 ± 2347.89	0.01	1.12	6.09777E-09	↑	1.52444E-07
GMP	971.01 ± 39.49	372.62 ± 19.00	0.01	0.38	1.24326E-19	↓	3.10815E-18
GDP	1130.45 ± 16.16	1307.64 ± 48.16	0.02	1.16	1.93635E-09	↑	4.84087E-08
GTP	2987.91 ± 3.77	3002.93 ± 3.34	0.01	1.00	1.55676E-08	↑	3.89191E-07
Ici	10469.79 ± 973.58	4671.24 ± 206.51	0.03	0.45	3.95202E-13	↓	9.88006E-12
AKG	11075.80 ± 47.38	11574.09 ± 181.36	0.03	1.04	1.20155E-07	↑	1.20155E-07
Lac	934260.38 ± 20965.59	1057345.85 ± 27962.64	0.03	1.13	1.66183E-09	↑	4.15458E-08
Mal	15582.01 ± 135.98	18769.20 ± 345.81	0.01	1.20	4.73888E-16	↑	1.18472E-14
NAD^+^	52.48 ± 6.44	87.80 ± 2.64	0.05	1.67	4.14575E-12	↑	1.03644E-10
NADP^+^	98.14 ± 10.94	201.36 ± 6.04	0.03	2.05	9.18086E-16	↑	2.29521E-14
3-PGA	79735.07 ± 2522.72	110915.54 ± 4602.53	0.04	1.39	2.83031E-13	↑	7.07577E-12
PEP	23402.16 ± 244.60	21629.80 ± 351.59	0.02	0.92	1.23629E-10	↓	3.09073E-09
Pyr	101096.33 ± 5695.47	41266.16 ± 3244.80	0.04	0.4	1.58494E-16	↓	3.96236E-15
Suc	7133.13 ± 224.57	5130.79 ± 228.65	0.01	0.72	1.18921E-13	↓	2.97303E-12
TPP	232.06 ± 5.64	260.05 ± 5.13	0.08	1.12	8.49886E-10	↑	2.12472E-08

(Data are presented as mean ± SD).

AMP, adenosine 5’-monophosphate; ADP, adenosine 5’-diphosphate; cAMP, cyclic adenosine monophosphate; DHAP, dihydroxyacetone phosphate; FMN, flavin mononucleotide; FBP, fructose-1, 6-bisphosphate; F6P, fructose6-phosphate; G6P, glucose6-phosphate; GMP, guanosine 5’-monophosphate; GDP, guanosine 5’-diphosphate; GTP, guanosine5’-triphosphate; AKG, alpha-ketoglutarate; NADP^+^, nicotinamide adenine dinucleotide phosphate; 3-PGA, 3-phosphoglyceric acid; PEP, phosphoenolpyruvic acid; Suc, succinate; TPP, thiamine pyrophosphate.

### 3.3 The results of the CCK-8 cell proliferation assay and senescence-associated SA-β-gal staining for the percentage of senescent cells demonstrated that H_2_O_2_-induced KGN cells exhibited significant proliferation inhibition

**Figure 2:**
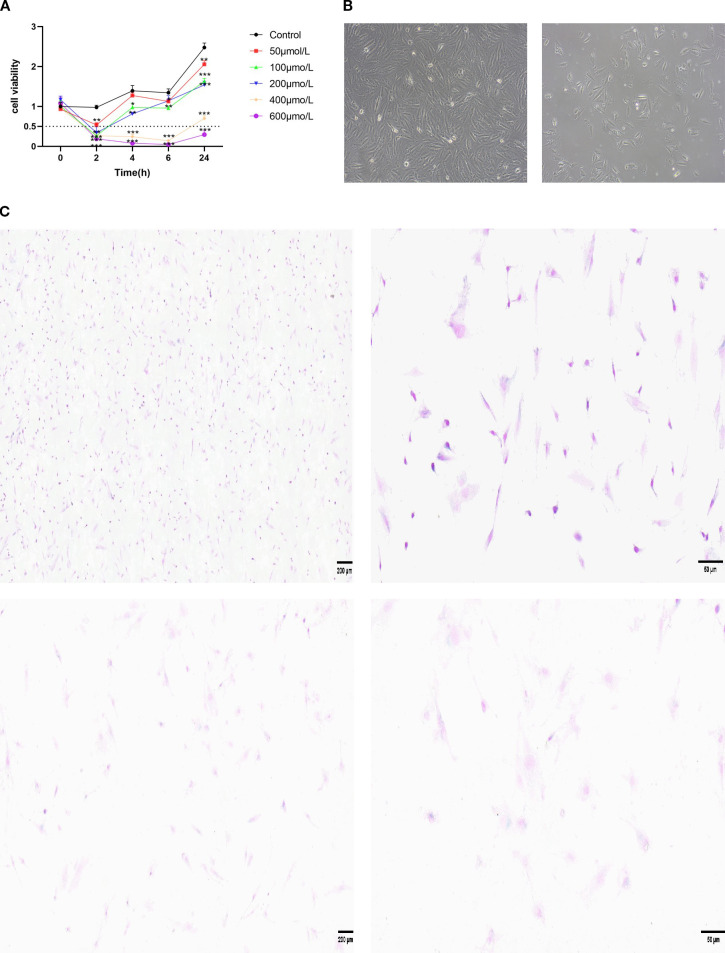
**(A)** Effect of different H_2_O_2_ concentrations on KGN cells viability as determined by CCK-8 assay. **(B)** Observations of the number and morphology of KGN cells in the control group and those treated with 50 μM H_2_O_2_ for 2 h. **(C)** SA-β-gal staining: control and H_2_O_2_ groups (scale bars: 200 μm, 50 μm). (*p < 0.05, **p < 0.01, ***p < 0.001).

Statistical analysis of CCK-8 data demonstrated highly significant main effects of time (F_4_,_60_ = 329.4, p < 0.001) and dosage (F_5_,_60_ = 349.9, p < 0.001), along with a significant dosage × time interaction (F_20_,_60_ = 45.7, p < 0.001), indicating a marked decline in cell viability with prolonged incubation and enhanced inhibition at higher concentrations, which suggests a synergistic interaction (**Figure 2A**). Tukey’s multiple comparisons test revealed significant inhibition beginning at 2 h (control vs. 50–600 μmol/L, p < 0.001), peaking at 24 h with all concentration groups differing significantly from the control (p < 0.001) and showing distinct inhibition intensities between adjacent groups (p < 0.001). Cell viability remained at 0.999 in the control group. In contrast, treatment with 50 μmol/L H_2_O_2_ for 2 hours reduced cell viability to 0.535, which corresponds to a decline in relative cell viability to 53.52% (inhibition rate: 46.48%) (p < 0.001). Electron microscopy analysis revealed that compared with the control group,treatment with 50μM H_2_O_2_ for 2 hours significantly inhibited KGN cell viability,which decreased toapproximately 50% (**Figure 2B**). Based on previous reports that senescence typically corresponds to a viability range of 50%–70%, reflecting proliferative arrest without nonspecific death from higher H_2_O_2_ exposure, a KGN cell senescence model was established using 50 μM H_2_O_2_ for 2 h to facilitate subsequent mechanistic investigation.

Following staining, five random microscopic fields were examined per replicate by an evaluator blinded to sample group assignments to prevent observational bias. The positive rate for each replicate was calculated as the mean percentage of positive cells across the five fields, yielding values of 5.62 ± 0.68%, 8.63 ± 0.88%, and 6.37 ± 0.50% in the control group, compared to 31.08 ± 4.22%, 34.79 ± 3.66%, and 33.23 ± 3.03% in the H_2_O_2_ group.An unpaired t-test with Welch’s correction was employed to compare the groups. The assumption of equal variances was tested using an F-test, which yielded F = 1.408 and P = 0.8305, indicating no significant difference in variances. The Welch-corrected unpaired t-test revealed a highly significant difference (t = 18.62, df = 3.888, P < 0.0001). The pooled mean was 6.87% for the control group and 33.04% for the H_2_O_2_ group, resulting in a mean difference of 26.16 ± 1.405% (95% CI: 22.21 to 30.11). The proportion of variance explained (eta squared, R²) was 0.9889. Effect size analysis indicated a very strong association, with r = 0.9944 and Cohen’s d = 18.89, demonstrating a powerful link between H_2_O_2_ exposure and increased cellular senescence. Morphological assessment of SA-β-gal staining revealed uniformly distributed light purple punctate or short rod-like structures in control cells, with a senescence-positive rate below 10%, consistent with baseline senescence levels. In contrast, H_2_O_2_-treated cells exhibited intense blue-purple staining indicative of moderate to severe senescence, a positive rate of 30%–40%, and sparse cell distribution attributable to H_2_O_2_- induced cell death or detachment (**Figure 2C**). Collectively, these results confirm that H_2_O_2_ successfully induced senescence in KGN cells.

### 3.4 Seahorse XF

Seahorse XF technology represents the established method for cellular energy metabolism assessment, quantifying mitochondrial respiratory function via OCR and glycolytic flux via ECAR. The experiment included two groups (control and H_2_O_2_) with three biological replicates each. Both ECAR and OCR were measured dynamically at 12 time points across a 120-minute period.

**Figure 3:**
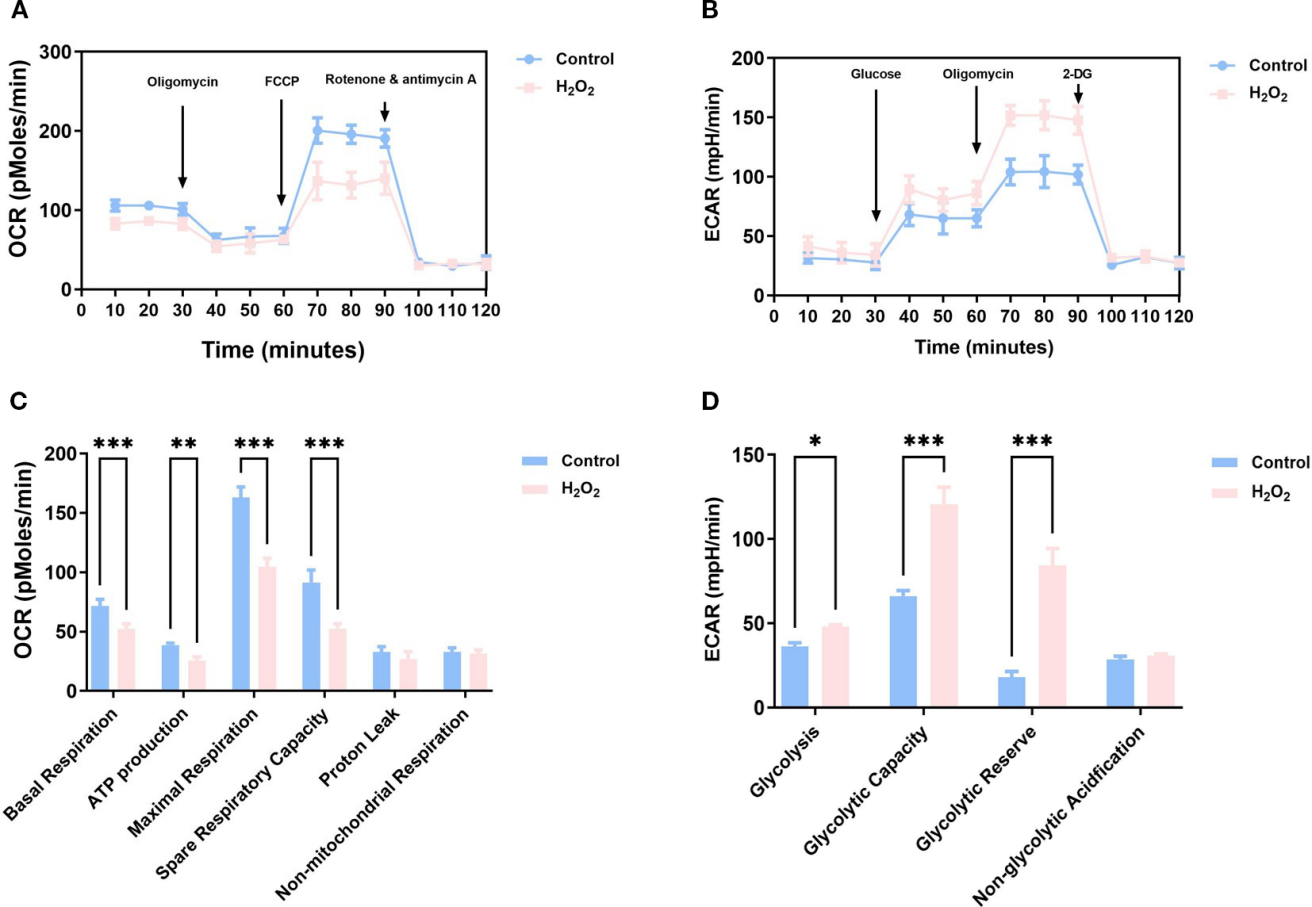
Assessment of ECAR and OCR in KGN cells. ECAR and OCR were assessed in control and H_2_O_2_-treated groups using the Seahorse XF Analyzer. **(A, C)** OCR measurements. **(B, D)** ECAR measurements. **(A, B)** Data are presented as mean ± SD, **(C, D)** subtracting non-mitochondrial respiration from the baseline OCR, *p < 0.05, **p < 0.01, ***p < 0.001).

OCR, a key parameter reflecting the efficiency of cellular oxygen utilisation, is a core indicator for assessing mitochondrial function under physiological conditions. In this study, oligomycin was employed to inhibit ATP-linked respiration by blocking mitochondrial ATP synthase, whereas the uncoupler FCCP was used to induce maximal respiratory capacity by collapsing the proton gradient; subsequent addition of rotenone and antimycin A enabled precise determination of non-mitochondrial respiration. The results demonstrated significant reductions in both basal and maximal mitochondrial respiratory capacity in H_2_O_2_-treated cells relative to controls (**Figures 3A, C**). RM-ANOVA revealed significant main effects of group (F_1_,_4_ = 20.03, P = 0.0110) and time (F_2_._588_,_10_._35_ = 200.3, P < 0.0001), along with a significant group × time interaction (F_11_,_44_ = 10.68, P < 0.0001). Estimated marginal means analysis further indicated that the overall OCR was significantly lower in the H_2_O_2_ group (77.64) than in controls (99.60), with a mean difference of 21.96 (95% CI: 8.34–35.59), and the inhibitory effect was most pronounced during the maximal respiration phase (70–90 minutes), where OCR decreased by 30.4%. Compared to the control group, the H2O2-treated group exhibited a significant reduction in basal respiration by approximately 27%. This impairment extended to ATP respiration, which decreased by about 34%, while maximal respiratory capacity was severely compromised by nearly 36%. Furthermore, the spare respiratory capacity was markedly diminished by approximately 43%, indicating a widespread and significant suppression of mitochondrial respiratory parameters (**Table 3**).

**Table 3:** Parameters related to ECAR and OCR in the control group and H_2_O_2_ group.

Parameters	Control	H_2_O_2_	Control - H_2_O_2_	95% CI	p-value
Basal Respiration	71.59	52.4	19.19	[9.42, 28.96]	<0.001
ATP production	38.68	25.41	13.27	[3.50, 23.04]	0.01
Maximal Respiration	163	104.7	58.28	[48.51, 68.05]	<0.001
Spare Respiratory Capacity	91.42	52.33	39.09	[29.32, 48.85]	<0.001
Proton Leak	32.91	26.99	5.921	[-3.85, 15.69]	0.22
Non-mitochondrial Respiration	32.72	31.61	1.11	[-8.65, 10.89]	0.82
Glycolysis	36.26	48.12	-11.86	[-21.36, -2.36]	0.02
Glycolytic Capacity	66.15	120.6	-54.4	[-63.90,-44.90]	<0.001
Glycolytic Reserve	18.03	84.29	-66.26	[-75.76, -56.76]	<0.001
Non-glycolytic Acidfication	28.54	30.87	-2.33	[-11.83, 7.17]	0.61

(Data are presented as mean).

ECAR quantifies the release of glycolytic end products (e.g., Lac) by detecting changes in medium pH; the ECAR elevation following glucose addition reflects basal glycolytic activity, subsequent oligomycin treatment reveals maximal glycolytic capacity, and 2-deoxy-D-glucose (a competitive inhibitor of hexokinase) suppresses glycolysis to confirm the glycolytic origin of the measured ECAR. As shown in the ECAR curve, compared with the control group, the glycolyticrate, glycolyticcapacity andglycolytic reserve of cells in the H_2_O_2_ group were increased, reflecting the enhancement of the glycolytic pathway in senescent cells (**Figures 3B, D**). Statistical results demonstrated a highly significant Group×Time interaction (F (11, 44)=7.400, P<0.0001), as well as significant main effects of time (F (3.547, 14.19)=150.9, P<0.0001) and group (F (1, 4)=56.09, P = 0.0017). The estimated marginal mean of ECAR was 57.00 in the Control group and 76.00in the H_2_O_2_ group, with a mean difference of -19.00 (95% CI: -26.05 to -11.96) between the two groups. Analysis of the derived glycolytic parameters revealed that, compared to the control group, the H_2_O_2_ group exhibited increased maximal glycolytic capacity and glycolytic reserve (**Table 3**).

Taken together, these results demonstrate that cells in the H_2_O_2_ group exhibited decreased OCR and increased ECAR compared to the control group. This metabolic shift indicates enhanced glycolysis alongside attenuated OXPHOS and TCA cycle activity in GCs following ovarian aging, preliminarily revealing a reprogramming of mitochondrial energy metabolism from OXPHOS towards aerobic glycolysis.

### 3.5 Kit for detecting Lac, Pyr, and glucose levels

**Figure 4:**
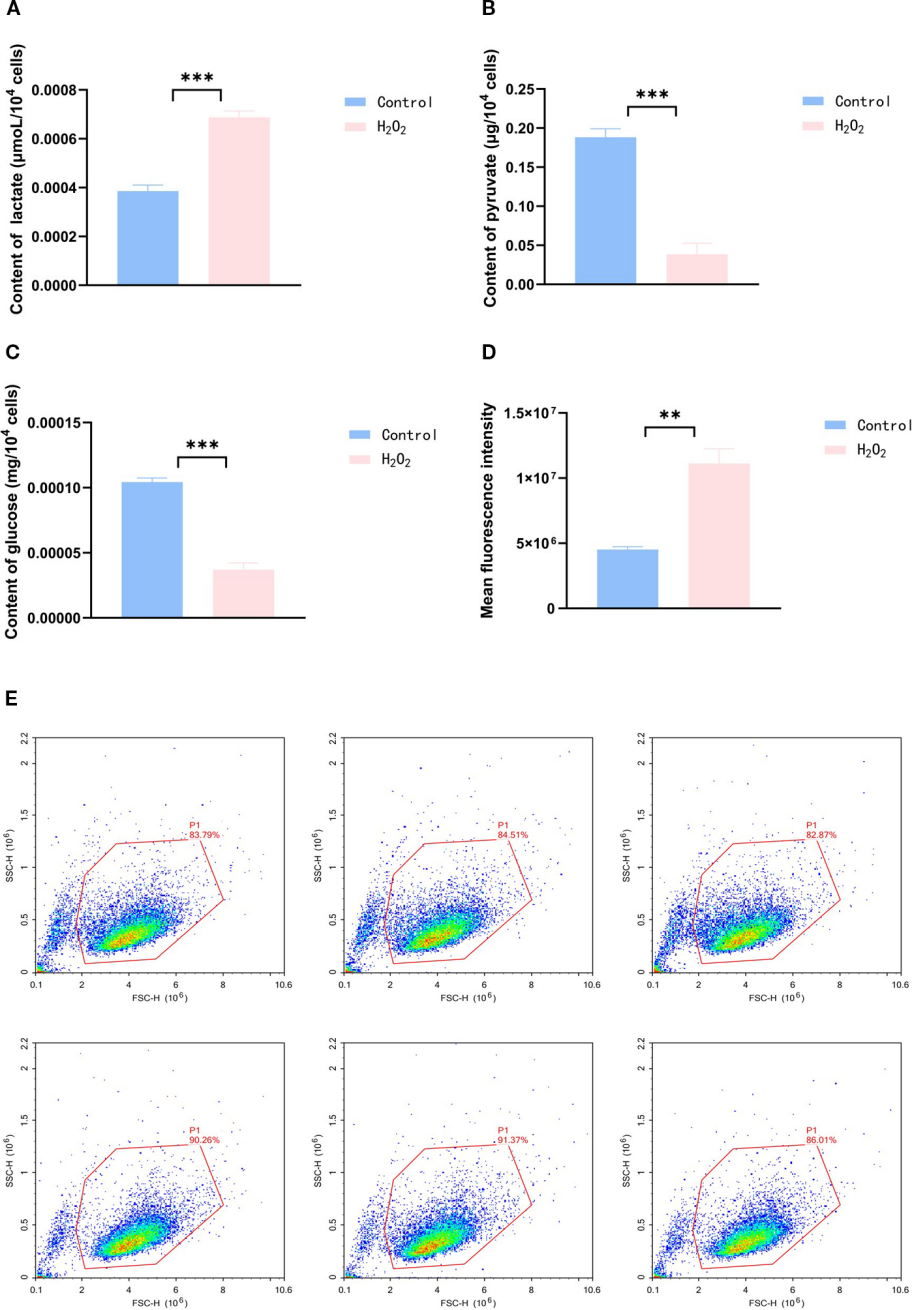
**(A–C)** Measurement of Lac, Pyr, and glucose levels in the control and H_2_O_2_ groups using kits. **(D)** Mitochondrial mtROS production rates in the control and H_2_O_2_ groups. **(E)** Flow cytometry analysis of mtROS in the control and H_2_O_2_ groups. (**p < 0.05, ***p < 0.01).

This study examined the effects of H_2_O_2_ treatment on key cellular metabolites (glucose, Lac, and Pyr). Group comparisons between control (n=3) and H_2_O_2_ (n=3) used Welch’s t-test with Benjamini-Hochberg FDR correction. H_2_O_2_ exposure significantly altered all metabolic levels (all FDR q < 0.01), showing markedly elevated Lac concentrations alongside significantly reduced glucose and Pyr levels (**Figures 4A–C**) (**Table 4**).

### 3.6 Measurement of intra-mitochondrial mtROS levels

Mitochondrial ROS levels were quantitatively assessed under identical experimental conditions. Statistical analysis revealed a pronounced increase in ROS levels following H_2_O_2_ treatment (P < 0.001), with the effect size (Cohen’s d = 2.84) indicating a substantial biological effect. This elevation in oxidative stress corresponds with the observed metabolic alterations in the H_2_O_2_-exposed cells (**Figures 4D, E**) (**Table 4**).

**Table 4:** Lac, glucose, Pyr and ROS changes in the control group and H_2_O_2_ group.

Metabolite	Control group	H_2_O_2_ group	95% CI	p-value	q-value
Lac	0.0004 ± 0.0001	0.0007± 0.0001	[0.0002442,0.0003599]	<0.01	<0.01
Glucose	0.0001 ± 0.0001	0.0001 ± 0.0001	[-7.780e-005,-5.663e-005]	<0.01	<0.01
Pyr	0.1882 ± 0.01076	0.0385 ± 0.0140	[-0.1789,-0.1205]	<0.01	<0.01
ROS	4,516,916 ± 218,836	11,114,959 ± 1,146,213	[3890110,9305976]	0.01	0.01

(Data are presented as mean ± SD).

## 4 Discussion

This study evaluated targeted glycometabolic profiles of GCs from infertile women with AMA. We further established senescenct KGN cells to investigate their morphology, metabolic reprogramming indices, and mitochondrial function. Our findings demonstrate mitochondrial energy metabolism reprogramming in aging ovarian GCs, highlighting its potential for identifying novel therapeutic strategies for AMA infertility.

**Figure 5:**
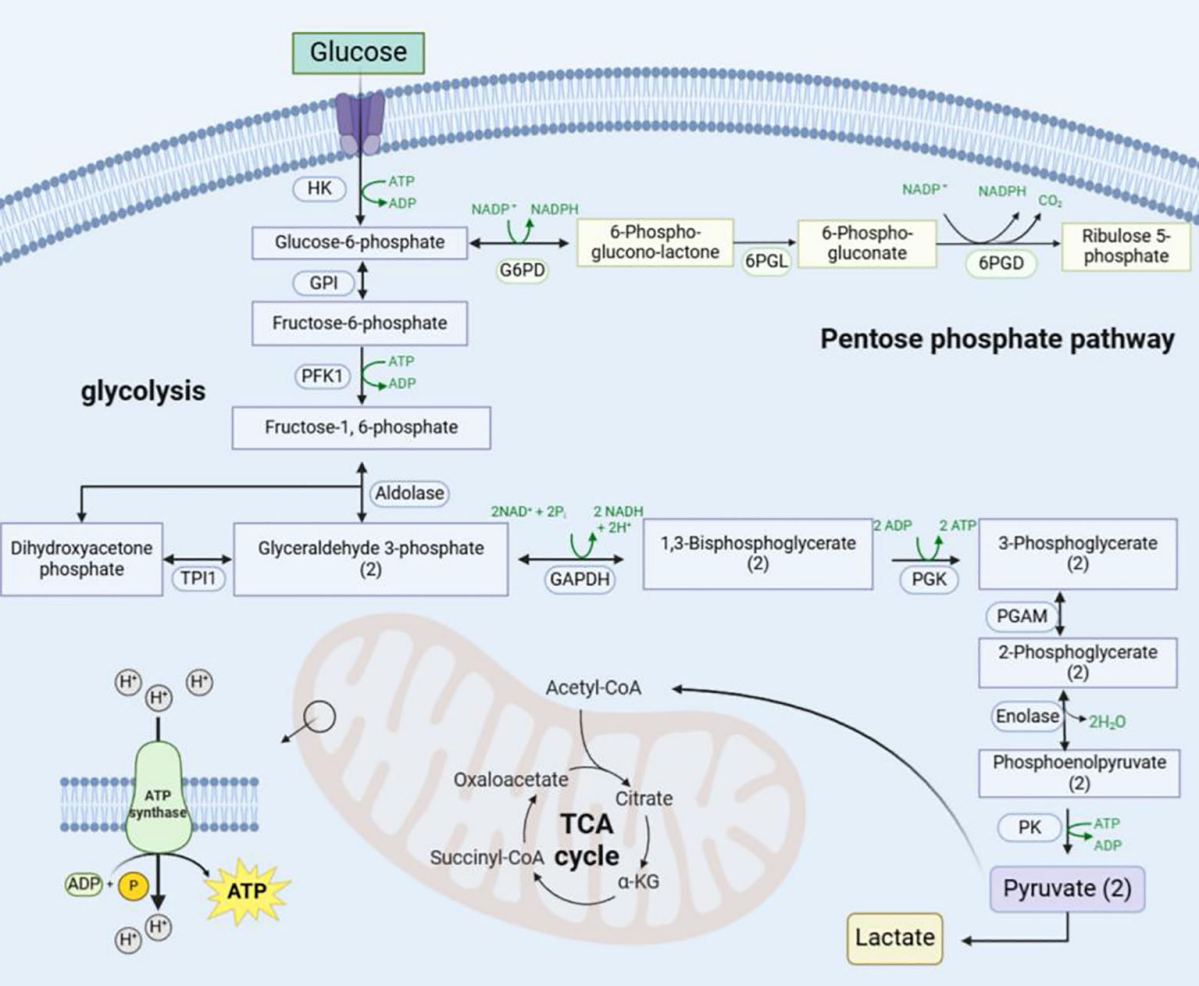
Schematic diagram of the mitochondrial metabolic reprogramming pathway. Glucose enters the cell via glucose transporter protein-1 and is converted to glucose-6-phosphate by hexokinase, which enters the pentose phosphate pathway catalysed by glucose 6-phosphate dehydrogenase on the one hand, and is converted to Pyr and Lac via the glycolytic pathway on the other. Later Pyr enters the mitochondria to be utilised and undergoes oxidative decarboxylation to generate acetyl-coenzyme A to enter the TCA cycle and OXPHOS. (“(2)” indicates that two molecules.) HK, Hexokinase; GPI, Glucose-6-Phosphate Isomerase; G6PD, Glucose-6-Phosphate Dehydrogenase; 6PGL, 6-Phosphogluconolactonase; 6PGD, 6-Phosphogluconate Dehydrogenase; TPI1, Triosephosphate Isomerase 1; GAPDH, Glyceraldehyde-3-phosphate dehydrogenase; PGK, Phosphoglycerate Kinase; PGAM, Phosphoglycerate Mutase; PK, Pyruvate Kinase; NADPH, Nicotinamide Adenine Dinucleotide Phosphate.

Glucose serves as the primary carbon and energy source for cellular growth and proliferation. Its metabolism encompasses the oxidative decomposition of glucose to generate energy for organisms and cells. The way of glucose metabolism is different under different physiological conditions. According to the different reaction conditions and reaction pathways, the way of glucose decomposition in the body can be divided into glycolytic pathway, pentose phosphate pathway, serine synthesis pathway, and mitochondrial TCA cycle (**Figure 5**). Glycolysis constitutes the central pathway, under normoxia, Pyr produced via glycolysis enters mitochondria for ATP generation through TCA cycle and OXPHOS (21). Initially described in cancer cells (Warburg effect), aerobic glycolysis replaces OXPHOS as the predominant metabolic mode, characterized by enhanced glucose uptake and preferential Lac production despite oxygen availability. Senescent cells exhibit similar mitochondrial metabolic reprogramming toward aerobic glycolysis. Beyond cancer, this effect occurs in septic immune cells (22), pulmonary arterial endothelial cells (23), fibrotic myofibroblasts (24), and ischemic-reperfused macrophages (25). In many types of cellular senescence, glucose metabolism shows different changes. Enhanced glycolysis promotes senescence in fibroblasts (26), while reduced neuronal glucose uptake occurs in Alzheimer’s microglia (27). Post-reproductive C. elegans shift from oxidative metabolism to anaerobic glycolysis, supporting metabolic reprogramming as a key senescence driver (28). Our targeted metabolomics reveals mitochondrial energy metabolism reprogramming in aging ovarian GCs, shifting from OXPHOS to aerobic glycolysis. This involves enhanced glycolysis alongside attenuated OXPHOS and TCA cycle activity. These changes are interconnected and tightly regulated by enzymes and metabolic intermediates. Mammalian cells utilize two ATP-producing systems (glycolysis and OXPHOS), adaptable to environmental changes (29). Under hypoxic conditions, glucose in the cytoplasm undergoes an enzyme-catalysed reaction and is converted to Pyr catalysed by Pyr kinase, of which hexokinase 2,phosphofructokinase(PFK)1, and Pyr kinase M2 are important rate-limiting enzymes of glycolysis (30), and Pyr no longer enters the mitochondria for TCA cycle but is dependent on Lac dehydrogenase A for its reduction to Lac. Normoxia permits Pyr oxidation to acetyl-CoA, driving TCA cycle and OXPHOS. Although glycolysis yields ATP faster than OXPHOS, its per-glucose ATP output is lower (31). OXPHOS and TCA cycle activity is inhibited with age in aging ovarian GCs and the glycolytic pathway is activated to compensate for the lack of ATP production (32).

Aging promotes glycolysis in GCs, and we observed increased glucose and Lac levels as well as decreased Pyr levels in GCs from the AMA group. Glucose uptake is the first rate-limiting step of glycolysis (33). The elevated levels of fructose 1,6-bisphosphate, fructose 6-phosphate, and glucose 6-phosphate in the AMA group corroborated enhanced glycolysis, indicating that high glucose utilisation is also evident in GCs of the aging ovary (34). During OXPHOS, Pyr is translocated to mitochondria via the mitochondrial Pyr carrier and converted to acetyl coenzyme A by Pyr dehydrogenase(PDH), which drives the TCA cycle to generate reduced NAD^+^ and reduced flavin adenine dinucleotide, supporting ATP synthesis (35). Under metabolic stress conditions such as hypoxia or oncogenic signals, PDH kinase levels are upregulated; PDH kinase inactivates PDH complex through phosphorylation, thereby shunting Pyr away from mitochondrial metabolic pathways and promoting glycolytic flux. Lac, once considered a metabolic waste product of anaerobic glycolysis, is now redefined as a pleiotropic molecule with emerging biological functions. Regardless of oxygen availability, Lac is an inevitable by-product of glycolysis (36) and can serve as a quantitative marker of enhanced glycolytic flux (37). In clinical diagnostics, Lac has also been identified as a diagnostic biomarker for various metabolic disorders. Pathological Lac accumulation is not only observed in cancer but also widespread in non-cancerous diseases (e.g., Alzheimer’s disease, heart failure, pulmonary fibrosis) (38). Meanwhile, Lac can function as an energy substrate through enzymatic cascade reactions. Under oxygen-replete conditions, Lac dehydrogenase mediates the bidirectional conversion of Pyr and Lac to achieve Lac recycling, while PDH imports Pyr into the TCA cycle through the irreversible acetyl-coenzyme A synthesis pathway, completing the full oxidation of Lac and ATP generation (39). Additionally, the conversion of Pyr to Lac is accompanied by NAD^+^ regeneration, which maintains the NAD^+^/reduced nicotinamide adenine dinucleotide ratio (40). Enhanced glycolysis would be expected to be accompanied by increased NAD^+^ levels in GCs from the AMA group. Interestingly, however, a causal relationship has been elucidated between decreased NAD^+^ levels and various age-related diseases (41). Previous studies have suggested that oocytes do not directly utilise glycolysis for energy supply, whereas GCs exhibit strong glycolytic activity from the primordial follicle stage onwards. The expression levels of PFK and Lac dehydrogenase in GCs are significantly higher than those in oocytes, with the strongest expression in antral follicles. The end products of glycolysis in GCs, Pyr and Lac, may serve as the main energy sources for oocytes. It has been found that adding Pyr and Lac to the medium for *in vitro* oocyte culture can significantly inhibit oocyte aging by improving the internal redox state and energy supply. Therefore, elucidating the regulation of Pyr and Lac production via the glycolytic pathway in follicular GCs is important for follicular development and oocyte growth.

The TCA cycle initiates with Cit formation from oxaloacetate and acetyl-CoA, culminating in CO_2_/H_2_O release and oxaloacetate regeneration (42). Metabolomic analysis revealed decreased Aco, Ici, and Cit levels in advanced-age GCs, indicating altered TCA cycle intermediates and overall suppression in senescent GCs.Cit is converted to Aco by aconitase (43). Age-related mtROS accumulation (44) and diminished mitochondrial membrane potential (45) inhibit aconitase activity, reducing Aco levels and disrupting TCA cycle flux. Functionally, Cit serves as a precursor for glutathione synthesis, providing glutamate to combat oxidative stress (46). It also modulates metabolic enzymes (e.g., PFK, PDH), balancing glycolysis and TCA cycle (47). Conversely, Mal activity increases post-aging, potentially as a compensatory mechanism (48). This metabolic rewiring may represent an adaptive response to mitochondrial decline, with dynamic Mal fluctuations serving as a potential biomarker for oxidative damage and TCA cycle remodeling during aging.

In the *in vitro* establishment of senescent KGN cells, H_2_O_2_-induced senescence was successfully achieved based on literature and preliminary data. CCK-8 proliferation assays identified 50 μM H_2_O_2_ (2-hour treatment) as the optimal condition, significantly inhibiting cell viability. Subsequent analyses confirmed: (1) reduced proliferation and increased senescence (SA-β-gal staining) in H_2_O_2_-treated cells; (2) Seahorse XF analysis showing elevated ECAR, suppressed OCR, and decreased OCR/ECAR ratio, collectively demonstrating a metabolic shift toward aerobic glycolysis.

Aging induces mitochondrial abnormalities—including rounded morphology, cristae disruption, and inner membrane loss—alongside reduced biogenesis and accumulated mtDNA mutations. These impairments diminish respiratory chain capacity, ATP production (49), OXPHOS efficiency, and elevate mtROS (50). Mitochondria, the most abundant organelles in oocytes, are closely linked to age-related quality decline. Dysfunctional mitochondria directly compromise oocyte competence (51), with age-associated infertility primarily stemming from reduced oocyte quantity/quality (52). Notably, aged oocytes exhibit decreased mitochondrial number/function (53), while transcriptome analyses of AMA oocytes reveal differential expression of mitochondria-related genes versus young controls (54). Preserving mitochondrial integrity is crucial for delaying ovarian aging, as redox imbalance from metabolic dysregulation and/or mitochondrial dysfunction underpins the pathophysiology of age-related infertility (55). When ROS overproduction overwhelms antioxidant defenses (56), oxidative stress triggers apoptosis in oocytes and GCs, detrimentally impacting female reproductive function.

Our flow cytometry analysis revealed elevated intra-mitochondrial mtROS levels in H_2_O_2_-induced aging KGN cells versus controls. Impaired electron transport chain function during metabolic reprogramming caused electron leakage, significantly increasing reactive oxygen species production; this redox imbalance triggers oxidative damage (protein carbonylation, lipid peroxidation, DNA breaks) that accelerates cellular senescence (57). In ovarian physiology, excessive mtROS impairs oocyte maturation, disrupts ovulatory cycles, and compromises luteal regression/maintenance, ultimately promoting GCs senescence and fertility decline (58). Furthermore, mtROS activates Sirt1/p53-mediated cell cycle arrest while inducing NF-κB-driven inflammatory SASP formation, amplifying senescence signals within the ovarian microenvironment (59). Thus, mitochondrial metabolic reprogramming, mtROS overproduction, and cellular senescence form a vicious loop driving ovarian aging and related pathologies (60).

## 5 Conclusions

In summary, our study demonstrates metabolic reprogramming in aging ovarian GCs characterized by enhanced glycolysis with attenuated OXPHOS and TCA cycle activity. Using an H_2_O_2_-induced oxidative stress model in KGN cells, we recapitulated glucose metabolic alterations observed in GCs of infertile women AMA. This work establishes the first experimental evidence linking mitochondrial energy metabolism reprogramming to ovarian aging. These findings provide a comprehensive framework for understanding age-related ovarian dysfunction and identify novel metabolic markers for future research.

Study limitations include unexplored relationships between Lac, Pyr and mitochondrial mtROS dynamics, whether Lac directly modulates GCs senescence, and its coordination mechanisms between glycolysis and OXPHOS—all requiring further investigation.

## Data Availability

The raw data supporting the conclusions of this manuscript will be made available by the authors, without undue reservation, to any qualified researcher.
